# Atelectasis in the Self-Prone Position in a Patient with COVID-19

**DOI:** 10.31662/jmaj.2021-0183

**Published:** 2021-12-08

**Authors:** Tomoki Mizuno, Jun Suzuki, Haruka Imai, Shiro Endo

**Affiliations:** 1 Department of Infection Prevention and Control, Tohoku Medical and Pharmaceutical University Hospital, Sendai, Japan

**Keywords:** Precordial atelectasis, COVID-19, obesity, awake self-prone positioning

A 44-year-old obese male (body mass index, 39.7 kg/m^2^) was diagnosed with moderate II coronavirus disease 2019 (COVID-19). His medical history included type 2 diabetes mellitus, hypertension, and hyperlipidemia. He was treated with remdesivir, oxygen, and awake self-prone positioning, which he performed for hours each day. Chest computed tomography for tachypnea at 6 days after onset revealed new precordial atelectasis in segment 3 on the right and left sides compared with the computed tomography results at day 2. Therefore, awake self-prone positioning was discontinued. Chest computed tomography after 5 days showed improvement in the atelectasis (Figure 1, arrows). Awake self-prone positioning is a useful strategy against the development of acute hypoxemic respiratory failure with atelectasis even for patients with COVID-19 ^(1), (2)^. Our patient’s atelectasis might have been associated with his excess weight in addition to excessive self-prone positioning. This case suggests that excessive self-prone positioning in obese patients induces atelectasis.

**Figure 1.**
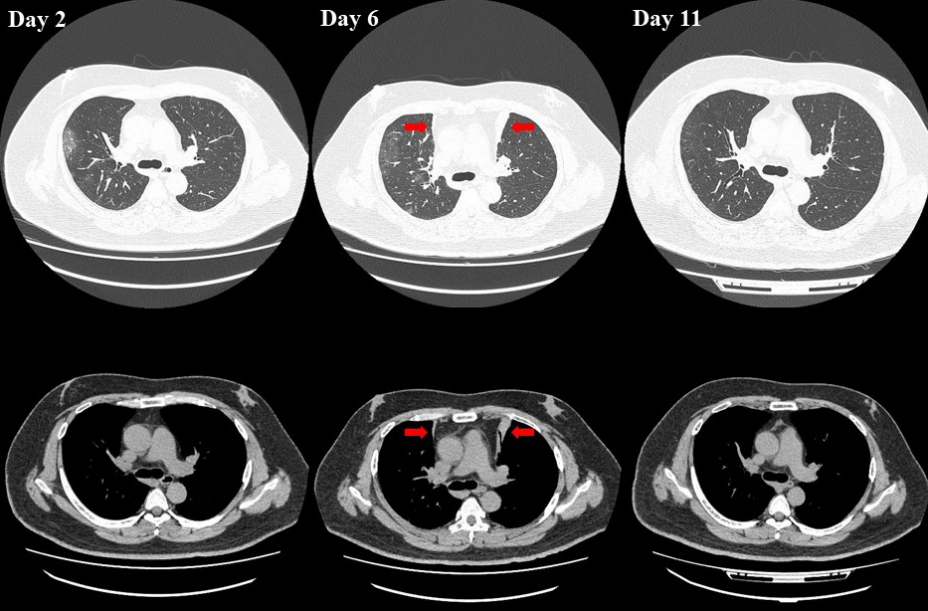
Results of chest computed tomography at 2, 6, and 11 days after onset.

## Article Information

### Conflicts of Interest

None

### Author Contributions

All authors contributed the design of the study. TM wrote the manuscript, and JS and SE helped to revise the manuscript. HI directly participated in the planning of the study. All authors read and approved the final manuscript. All authors meet the ICMJE authorship criteria.

### Approval by Institutional Review Board (IRB)

This study did not require IRB approval.

### Informed Consent

The patient provided written informed consent for publication of this report.
